# ‘Whose failure counts?’ A critical reflection on definitions of failure for community health volunteers providing HIV self-testing in a community-based HIV/TB intervention study in urban Malawi

**DOI:** 10.1080/13648470.2015.1077202

**Published:** 2016-01-13

**Authors:** Rodrick Sambakunsi, Moses Kumwenda, Augustine Choko, Elizabeth L. Corbett, Nicola Ann Desmond

**Affiliations:** ^a^ Malawi Liverpool Wellcome Trust Clinical Research Programme, Science Communication, PO Box 30096, Chichiri, Blantyre, Malawi; ^b^ College of Medicine, University of Malawi, Blantyre, Malawi; ^c^ Malawi Liverpool Wellcome Trust Clinical Research Programme, Blantyre, Malawi; ^d^ London School of Hygiene & Tropical Medicine, London, UK; ^e^ Liverpool School of Tropical Medicine, Liverpool, UK

**Keywords:** failure, HIV self-testing, community health worker, community counsellor, volunteerism

## Abstract

The category of community health worker applied within the context of health intervention trials has been promoted as a cost-effective approach to meeting study objectives across large populations, relying on the promotion of the concept of ‘com-munity belonging’ to encourage altruistic volunteerism from community members to promote health. This community-based category of individuals is recruited to facilitate externally driven priorities defined by large research teams, outside of the target research environment. An externally defined intervention is then ‘brought to’ the community through locally recruited community volunteers who form a bridge between the researchers and participants. The specific role of these workers is context-driven and responsive to the needs of the intervention. This paper is based on the findings from an annual evaluation of community health worker performance employed as community counsellors to deliver semi-supervised HIV self-testing (HIVST) at community level of a large HIV/TB intervention trial conducted in urban Blantyre, Malawi. A performance evaluation was conducted to appraise individual service delivery and assess achievements in meeting pre-defined targets for uptake of HIVST with the aim of improving overall uptake of HIVST. Through an empirical ‘evaluation of the evaluation’ this paper critically reflects on the position of the community volunteer through the analytical lens of ‘failure’, exploring the tensions in communication and interpretation of intervention delivery between researchers and community volunteers and the differing perspectives on defining failure. It is concluded that community interventions should be developed in collaboration with the population and that information guiding success should be clearly defined.

## Introduction

This paper is based on the findings from an annual evaluation of community health volunteers employed as community counsellors to deliver semi-supervised HIV self-testing (HIVST) at community level as part of a large HIV/TB intervention trial conducted in urban Blantyre, Malawi. Through an empirical ‘evaluation of the evaluation’ we critically reflect on the position of the community volunteer through the analytical lens of ‘failure’, and highlight the tensions and challenges that community volunteers face when involved in community-based intervention trials.

Community health workers (CHW) are increasingly relied on to deliver community-level interventions in a range of settings across sub-Saharan Africa (SSA) (Brugha et al. 2010; Mueller et al. 2011). In Malawi, this is, in part, due to the shortage of health personnel across the health system (Mueller et al. 2011), despite a significant focus on task shifting across primary and community health services (Brugha et al. 2010).

A combination of funding constraints and increasing recognition of the importance of community engagement in optimising the outcomes of intervention research and ensuring the application of ethical principles (Emanuel et al. 2004; Dickert and Sugarman 2005; Angwenyi et al. 2013) has led to the adoption of CHW in large community-based health intervention trials as community volunteers, often in combination with a cadre of health workers more formally employed within the trial. The role of these volunteers varies according to the trial objectives but commonly they are often quasi-employees and an important, and often large, group within the research team, although rarely are they formally employed. Similar to community development workers, reliance on their willingness to ‘volunteer’ is informed by the expectation that, because they are based within their home communities, they will also be able to maintain alternative sources of income. It is also informed by the ‘global ideology of sustainability’, which Swidler and Watkins (2008) describe as underlying increasing reliance on volunteers within ‘development’ projects. Swidler and Watkins (2008) highlight the ‘failure’ of those leading donor projects to recognise the materiality of life in resource-poor settings, a fact reflected in contemporary medical research conducted across SSA and highlighted elsewhere as an ethical concern (Maes 2012; Takasugi and Lee 2012). This materiality impacts differently on formally employed fieldworkers and community volunteers. In the latter case, a lack of permanent job status and consequent financial insecurity is exacerbated and may contribute to a greater degree of ‘failure’. Researchers also rely on perceptions of ‘community belonging’ to encourage altruistic volunteerism to promote community well-being and improve health (Flaherty and Kipp 2004). Reasons for volunteering within a community setting are often much broader, however, and reflect a wider range of functional motivations, including those of altruism, but also to promote individual ambition to progress, either through learning new skills, enhancing social position or access to social capital and reputation within the community or as a route to acquiring permanent employment status (Kaler and Watkins 2003; Swidler and Watkins 2008; Akintola 2011; de Wet 2011; Takasugi and Lee 2012; Seabe 2014). In Malawi, Chawanangwa et al (2005) suggest that community care givers are motivated by both intrinsic (empathy, altruism, religious conviction) and extrinsic factors (including community recognition, future employment hopes, access to financial resources) but that extrinsic factors are much less frequently mentioned by volunteers (Chawanangwa et al. 2005). This may reflect a social desirability bias in responses to questions concerning motivation and they conclude that materialities need to be highlighted in developing strategies to improve sustainability of practice amongst caregivers.

This paper shows how the multiple positionings of community counsellors between researchers and communities led to contradictory roles and expectations.

As ‘bridges’ between the researchers and the community, community health volunteers working within the context of a health intervention trial may often be perceived as cultural and scientific interpreters, informed by the tacit expectation to translate scientific knowledge to encourage trust, participation and uptake of the intervention, facilitated by shared ethnicity, language, socioeconomic status and life experiences. They are tasked with delivering ‘culturally appropriate’ services as cultural mediators (Geissler et al. 2008). Their roles within health intervention trials may be diverse, including interpretation and translation, help in sensitising communities with appropriate health education and information, assisting people in receiving the care they need, providing informal counselling and guidance on health behaviours, advocating for individual and community health needs, and providing other direct services such as first aid and blood pressure screening (Lehmann and Sanders 2007). Whilst their roles may vary, in charging volunteers with such central roles in framing community development, those coming from outside directing intervention practice often fail to acknowledge the complexities they introduce in the lives of community health volunteers. These volunteers are required to manage, negotiate and navigate a series of multiple and often conflicting socialities, relationalities (Geissler et al. 2008) and expectations driven by their interstitial position between insider/community member and outsider/representative of the intervention group. Representing (often conflicting) values, responsible for ongoing and informal information and education of their peers in support of the intervention aims, community volunteers are challenged to negotiate novel and externally-supported positions of power in their community, maintained only as long as they remain faithful to and driven by the needs of the research. Thus, whilst the community may view them as ‘experts’ on the intervention and rely on them to provide ethical and value-free advice regarding decisions to participate in the intervention, their position as respected community volunteers amongst their community is contingent on their ability to conform to external priorities, which may conflict with the position of trust ascribed to them by the community as a result of this role. Negotiating these conflicting expectations between the target community and intervention team is a substantial, but often unacknowledged, challenge of the role of community volunteers within the context of intervention trials.

Whilst scientific progress in medicine has been driven and informed by its failures as much as by its successes, publications of success have been much more prevalent in the scientific literature than those reporting failed trials. This has been largely due to a lack of resources and other motivations to write up negative trial results and lack of interest from journals in publishing these results (Lehmann and Sanders 2007). This trend is slowly changing to reflect the recognition that reporting reasons for the failure of interventions in health may help to prevent similar failure in future research at an earlier stage (Abderson and Doherty 2005; Coalition Against Major Diseases 2010; McGoey 2010; Kasenda et al. 2012). In contrast, failure has been explored in other scientific disciplines such as those of technology (Saetnan 1991; Torrens 1992; Gooday 1998; Downer 2009) and organizational theory (Arifio and de la Torre 1998) as well as information systems (Brown and Jones 1998; Bartis and Mitev 2008). This literature points to the role of people rather than the technology in the failure and that failure is relative, suggesting that how failure is perceived can depend on the beholder. Our research suggests that CHWs as volunteers can be set up to fail by the ways in which they are positioned in the interventions, which calls for an important overhaul in the ways in which these positions are institutionalised.

## Background

### The social context of urban Blantyre

Malawi is one of the poorest countries in the world with a Gross Domestic Product (GDP) per capita of US$270 and a population size of 16 million, with 50% below the national poverty index. Life expectancy at birth is 54 years and the under 5 mortality rate is 77 in every 1000 live births (World Bank, 2013 data). The country is divided into three regions, each with a regional capital. Blantyre City forms the regional capital of the Southern Region, with a population of just over 1 million in 2015. Whilst there is a growing middle class in urban centres, the majority of the urban population live in high density, slum areas in closely-packed houses, many of which are rented on a temporary basis, with shared toilet facilities and water sourced through community pumps, reflecting high rates of both in and out-migration, often between the city and rural homes.

### The HitTB intervention

It is against this background that the HitTB (*lit*. hit tuberculosis) intervention was developed ‘to investigate whether substantial declines in TB incidence can be achieved in a high HIV prevalence setting within a short time frame by a TB case-finding intervention combined with intensive HIV/TB prevention through community-level and home-based HIV self-testing and targeted prevention of HIV-related TB through a cluster-randomised trial’. The catchment area for the trial was purposively selected according to both population density and magnitude. Three main catchment areas in urban Blantyre with a combined population of 108,000 adults were selected and classified into 28 distinct clusters through a rigorous mapping activity (MacPherson et al. 2013). Clusters of 1200 adults were randomly assigned to control or intervention with ‘TB case notifications as the main outcome measure and aiming for a 35% difference between trial arms’.

Supervised and semi-supervised models for delivery of HIV self-testing (HIVST) were developed (Choko et al. 2011). These models were based on the level of counsellor support required by individuals taking part in the study and were delivered by community counsellors who also linked participants to facility-led distribution of cotrimoxazole and isoniazid preventive therapy as pre-ART HIV care for those who disclosed. The ability to measure effect was principally determined by a sufficiently high uptake of HIVST; thus, the role of the community counsellors was central to the success of the trial. A parallel system for community event reporting was designed through a number of cluster representatives reporting to a centrally employed Senior Community Liaison Officer. This aimed to ensure, in the words of the intervention, ‘ethical and responsive practice through establishing and maintaining a continuous community dialogue’ throughout the provision of HIVST kits within urban communities.

### Profiling community counsellors

A total of 28 community counsellors (14 men and 14 women) were recruited from the 14 intervention clusters. Their role was to provide, on a semi-voluntary basis, home-based HIVST services to residents from within their own clusters. Defined formerly as volunteers, the community counsellors received initially a minimal stipend of MWK 2000 (equivalent to approximately US$11 in 2012) per calendar month, with the expectation that they would have time to continue their other income earning activities. This ‘stipend’ was later increased to MWK 14,000 (equivalent to approximately US$56 in late 2012) following official devaluation of the local currency in 2012 (Dionne and Dulani 2013) and after a number of petitions put forward to research supervisors by the community counsellors themselves. This new figure was at the time still less than a monthly Ministry of Health salary for formal and full-time community health workers, Health Surveillance Assistants (HSA).

These volunteer community counsellors came from all walks of life, with a range of diverse occupations including housewives and those running small businesses. A number had been school dropouts and the majority were economically and socially equivalent to the communities they represented, although most possessed a Junior Secondary Certificate (equivalent of GCSEs). Counsellors were trained following the then current Malawi national HSA training programme. This programme draws on nationally appropriate rights discourses and contains components targeting HSA roles with respect to national well-being, situating community health workers, recognising their positionality and emphasising their obligation as community workers (Kok and Muula 2013).

### Community-led processes of volunteer recruitment in HitTB intervention

Volunteer Community Counsellors were identified and selected during community sensitisation meetings, organised by the research team to introduce each community to the HitTB HIV/TB intervention trial. Meetings were conducted in all 28 intervention and control clusters, facilitated by the research team with the help of locally identified leaders and catchment HSAs. The selection process was designed to ensure individuals were recruited through a fair and democratic process. Before selection, community members were provided with guidelines by the researchers, introducing criteria to consider before nominating a candidate. The guidelines were prescribed according to the needs of the intervention trial to ensure effective implementation and promote ethical representation of all community members resident in each catchment area. This ostensibly ensured that individuals identified were suitable for both the research team and the community. Specific criteria were that a counsellor had to be: (1) a usual resident of the catchment area (defined as sleeping in the area for more than 2 weeks in every month), (2) willing to volunteer, (3) an individual who was dependable, respectful, accessible, honest and be able to maintain confidentiality. Names of individuals, both present during the meeting or in *absentia*, were proposed by community members and a general vote conducted. Local leaders and HSAs were responsible for counting votes and consolidating the results in full view of all community members present at the meeting.

### Roles and responsibilities of voluntary counsellors within the community

Community counsellors played a central role in community provision of HIVST services, driving uptake within their cluster and providing the main link between the community and the research. They were responsible for ensuring that all adult cluster residents had access to HIV testing services, including appropriate access to pre and post-test counselling and the provision of information on instructions for use for HIVST kits. They were also required to conduct TB screening for all clients requesting HIVST services. Clients were able to self-present to the counsellor's house, undergo test to assess their ability to conduct the test themselves using a dummy kit (‘competency test’) and take the HIVST kit with them to test individually or as a couple at home, or self-present for semi-supervised HIVST at the counsellor's home. Alternatively, counsellors were also requested to provide home-based HIVST within their cluster, moving from door-to-door to introduce and explain the offer of HIVST. Regardless of testing modality, clients were in all cases requested to return the used test kit within a sealed envelope provided by the counsellor and posted into a ‘ballot box’ stored in an accessible location at the counsellor's residence. These boxes were regularly emptied and anonymised, used kits were delivered for laboratory validation and quality assurance of test results. Voluntary community counsellors were required to maintain a rate of between 8 and 16 people aged between 16 and 49 tested per day of the intervention period of 2 years. Counsellors were also expected to facilitate linkage to appropriate cascades of care for TB and HIV, linking clients to facility-based services. It was estimated that counsellors should spend between 30 and 50 minutes per client, working 8 hours a day, 6 days a week and conduct not less than 96 tests a week. Whilst they were required to attempt equal representation by gender, targeting men was acknowledged to be difficult since most working men generally finished work at 5.30pm and were often home by 6.00pm thus forcing community counsellors aiming to reach men in their community to work late or on Sundays.

Throughout their role, counsellors were expected to maintain confidentiality at all times to ensure continued trust in the HIVST service and optimise uptake. Monitoring was facilitated through community supervisors who visited the counsellors on a regular basis, providing a route to communication with the research team. Each counsellor maintained a testing register in which they recorded socio-demographics and addresses of those who had tested, although not test results. This register was crosschecked regularly against stock supplied to each counsellor and this stock was replenished frequently to ensure the constant availability of kits, seven days a week. In addition, community counsellors were required to produce weekly uptake reports detailing numbers of men, women and couples who had tested. They were also required to report any adverse events occurring as a result of using the test kits. Such adverse events included those related to the test kit such as faulty readings as well as more social impacts such as incidents of violence either in coercive testing or following disclosure. In order to maintain their reputation and sustain awareness of the availability of HIVST in the cluster community, counsellors were required to constantly sensitise the community through the distribution of flyers and regular interaction with potential clients. Given this need to sustain continued community interest in HIVST within their cluster, and despite their role as ostensibly, voluntary and unpaid, community counsellors were charged with a large amount of work to meet the requirements of the intervention. Table 1 highlights the range of tasks required of voluntary community counsellors in the provision of semi-supervised HIVST.

**Table 1.** Responsibilities of voluntary counsellors offering HIVST in urban Blantyre

*Pre-testing*
1. Booking clients in advance as appropriate and pursuing opportunities for encouraging uptake of HIVST.
2. Provide self-presenting and home-based clients with verbal information, leaflet and information sheet.
3. Record the booking on the HIV testing register.
4. Provide a barcode for each client and place stickers on all forms – keep remaining stickers for use if required (TB suspect and other referral).
5. Keep alphabetical file of all loose forms once completed.
6. Review information sheet with client prior to testing and request two signatures for consent (one for the client and one for the counsellor file).
7. Document consent in the testing log.
8. Conduct TB symptom screening and record possible suspects in Cough Register. Add a matching client barcode to the TB microbiology form and fill in the client name and barcode on two sputum cups.
9. Explain to the client how to collect sputum and request they return the form and cups to research team members at the local health facility.
10. Conduct a competency test if the client requests to self-test in private or at home.
11. Explain the procedure for collection of oral mucosal transudate (OMT) with the spatula and review the test algorithm with the client.
12. Provide the client with the HIVST kit, self-completion exit interview and envelope for return of used kit, explaining how to complete the exit interview.
13. Record the HIVST kit number against the client identifier within the testing register.
14. Provide pre-test counselling and emphasise the need for post-test counselling and returning the kit within 24 hours.
15. Complete all pre-test sections of the register and self-referral card for the client to take to a local facility for follow-up.
*Post-testing*
1. Ask the client if the test was successful and about their test results, requesting to see the kit if the client allows.
2. Offer to repeat the test if found to be positive or invalid.
3. Provide results-based post-test counselling.
4. Advise the client to post the used envelope containing the kit in the ballot box.
5. Complete the reverse of the referral card and the testing log and register.
6. If a positive result, facilitate appropriate referrals with supporting forms and advise the client who to see at the local facility.

This paper has developed from a pragmatic assessment of trial implementation. All authors were involved in the HitTB study: MK was responsible for the social science research component, AC for the data component whilst EC was the overall Principal Investigator and ND provided technical lead on the community, social science and field design. RS was the Community Liaison Officer (CLO) ‘within’ the intervention; this provided him with an insider's perspective since he was responsible for all field activities, including monitoring and supporting volunteer counsellors while at the same time listening and responding to community concerns. The CLO performed these roles over 3 years, which cemented a long-term relationship between him, the research team and the counsellors with regular informal and work-related meetings, discussions of concerns and insights into their lives. There was good rapport between RS and the volunteers, who confided to him openly about their problems regarding the study during the evaluation interviews. In this role RS had full access to all project meetings and was also able to observe the formal process of the trial. He maintained regular notes of his observations and these insights gave rise to the need to analyse the relationships further. Accurate evaluation was facilitated by a strong and trusting relationship between the evaluators and counsellors; influencing the planning and design of the process as well as shaping the flow of the interaction during the evaluation itself and ensuring counsellors felt free to express their opinions during the process.

### Methods: the assessment process

Upon recruitment, volunteer counsellors were warned that their work and uptake figures would be monitored regularly throughout the intervention period through weekly uptake reports submitted to the research team and feedback was provided on a regular basis throughout the year when a shortfall in uptake was identified, addressing community counsellor concerns throughout their period of service and supporting them to meet their weekly targets.

Whilst monitoring was conducted throughout the period of HIVST provision, after one year the Trial Coordinator and Community Liaison Officer within the research team conducted an evaluation of community counsellor practice to assess individual progress of each counsellor in meeting his or her objectives. Similar to the selection process for community counsellors, the evaluation was designed to promote openness, transparency and full participation. Specifically, the evaluation assessed protocol adherence and weekly uptake rates of individual counsellors in order to predict whether or not the trial would be likely to meet its uptake targets (over 80% of cluster adult population tested) within the study timeframe. This paper draws its findings from results that arose from this evaluation that identified the need for an in-depth analysis of the roles and expectations of volunteer community counsellors and a significant overhaul of the ways in which the expectations of the researchers were met.

This paper is based on one-off evaluation sessions that included multiple research tools. RS was also part of the team who designed the evaluation process including both quantitative and qualitative data collection tools. These evaluations were designed to evaluate counsellors in relation to their performance ‘for’ the intervention but the critiques they raised during these sessions quickly turned them into meetings where they were able to report the challenges they faced in their roles.

A total of 28 individual interviews were conducted with counsellors at sites in each counsellor cluster and which lasted for approximately 90 minutes each. These in-depth discussions focused on themes ranging from individual performance assessment against original set targets to meeting uptake rates and communities’ perceptions of counsellors’ conduct in service delivery. Interviews also included discussions around the number of tests each counsellor had been able to conduct, precision in filling out forms and registers, adherence to protocol guidelines, general self-conduct and communities’ observations and recommendations reported through an embedded community liaison system, community perceptions of how they played their role were highly valued. In addition, information was collected through role-play exercises of mock HIVST sessions to explore how each counsellor managed each step in the intervention process and to ensure that no steps were omitted in service delivery. At the end of each interview the community counsellor appraised his/her own performance and compared this to the assessment conducted by the Trial Coordinator and CLO. Responses and discussions were individually rated by both the Co-ordinator and CLO. On completion of the evaluation, each counsellor was provided with an individualised verbal and written report of their performance and given the opportunity to review it and respond to the feedback. All the interviews were conducted in the local language ‘Chichewa’, and all data were recorded on questionnaires with additional notes taken. The information, including quotations, included in this paper was collected from reports compiled at the end of each formal interview.

The final component of the evaluation was a document review that involved physically checking all registers and other documentation used during daily work routines to check completion and storage conformed to the protocol. Other documents checked were HIV testing diaries and logs, participant consent forms, TB screening forms, care referral forms and pre- and post-test counselling registers.

## Results

### Summary of the evaluation results

A comprehensive performance report was compiled detailing the process, findings, views of community counsellors and recommendations from community members. In total, 26 of the 28 community counsellors were found to have performed well over the previous 12-month period, each achieving over 80% adult uptake of HIVST within their cluster. The qualitative components of the assessment additionally highlighted ethical conduct in their role performance, particularly in terms of confidentiality, accessibility, behaviour towards clients, commitment to their work and their clients and general good conduct, with no serious concerns raised by community members. Further, these 26 individuals had maintained adequate record keeping and protocol adherence throughout service delivery. The research team unanimously approved to renew contracts for a further 12 months for the 26 successful counsellors. When offered renewal contracts one individual declined to renew, citing other commitments preventing his continuing in this role. This individual had been one of the leading performers and the team agreed that it was worth exploring why he had decided to end his contract. Alluding to more tensions to come, the enquiry revealed that he had alternative, more lucrative, sources of income that were increasingly difficult to maintain in the face of the demands of the project.

The two remaining counsellors were deemed, during the assessment process, to have failed to achieve their targets and performed badly during the appraisal process.

The minimum requirement was to test not less than 80% of the adult population in their respective clusters. They had each managed to reach an average of 67%, by the end of 12 months period. Each had also scored below standard on protocol adherence, and each was found to have deliberately offered self-testing kits to clients who were living outside the demarcated cluster boundaries, although it was not clear why this had occurred. Each had also failed to subscribe to the pre-defined service protocol, particularly in offering test kits to under-age individuals (<16 years old). They had also recorded incorrect data in the registers to hide these protocol errors. Compounding the assessment on uptake, community reports through the community liaison system identified concerns of breaches in confidentiality. It was agreed through the appraisal that the contracts for these two counsellors would not be renewed, and they were defined as having failed.

### Unpacking failure: community counsellor perspectives

Whilst both community and institutional reviews were consistent in identifying counsellors who had failed to meet performance expectations, the evaluation process was designed to ensure ethical practice and community counsellors were provided with the opportunity to exonerate themselves before a decision was made to declare them as failures. Interestingly, and despite the evidence to the contrary, both counsellors insisted that they had not failed in their performance but had, rather, managed to achieve when faced with a number of challenging constraints. Amongst their arguments were:

1. Emphasis on the need for non-coercive and voluntary testing, which translated to a reliance on self-presentation, and many adults residents in their clusters failed to come forward.
2. Emphasis on ethical practice and the patience to spend time with each individual presenting to test the reduced number of individuals they were able to test.
3. Emphasis on the additional obligations they felt towards their families, which required them to pursue more lucrative income opportunities with their time.
4. Emphasis on unreachable targets which could not be attained without coercion.

Both failed counsellors were men who felt pressured to pursue a number of income earning strategies concurrently and whilst they had accepted their nomination by the community, in recognition of the implicit respect it represented, they were challenged to maintain uptake. The voluntary nature of the role combined with the need for high levels of uptake, meant that it was difficult but necessary to combine routine economic activities with the demands of the intervention. Stipends were felt to be wholly inadequate and although these incentives were revised upwards during the intervention period, the amount provided within the first year provided insufficient income to enable counsellors to focus wholly on their ‘voluntary’ role. Tension between everyday income strategies and the time required to meet targets was described by many of the counsellors during evaluation interviews, in the first example by a female community counsellor who earned her usual income through the second-hand clothes business and cited this to explain periods of unavailability.

Well, sometimes I have been unavailable in my cluster because I go to Limbe market to order some second hand clothes for my small business. You know the money that we are paid at the end of the month isn't enough to support my family, that is why I still do my ‘Kaunjika’ [second-hand clothes] business. I would have completely stopped this business if this counsellor job was a full time job, I mean if we were being paid good money at the end of the month.

In the next example, a male community counsellor raised the issue of voluntariness and being asked to work part-time versus full-time in the intervention trial as follows:

When they were engaging us in this job they told us that we are volunteers, we understood what that meant and we accepted that, but now they tell us to work full time, how can they say we should work for 8 hours a day 5 days a week, sometimes 6 days a week, and still expect us to be volunteers? This is becoming difficult for me because I can't do other businesses that my family depends on for survival.

Assurance of ethical practice in the provision of HIVST within the community was emphasised during community counsellor training. However, over time and in order to meet the pressure of weekly targets for cluster level uptake figures, counsellors were increasingly challenged to balance their remit to ensure they avoided coercive testing and their remit to ensure targets were met. The challenges in balancing these two conflicting requirements are described in the following examples taken from two female community counsellor interviews:

I really tried hard to offer testing to all the adults in my cluster; you know it's not my problem that I can't test enough people. This is research and in HIV testing you cannot force anybody to test, you can only ask them to take part, if they don't want to then you can just try to a point to persuade them but not forcing them, so it is wrong to say I haven't reached the target.

One area of my cluster is full of Apostolic faith people and these people do not take part in anything to do with medicine, they don't take medicine, they don't go to the hospital and they say they can't test for HIV, and this has taken up part of the population of my cluster, as a result it would have been difficult for me to test many people.

On one hand the quotes demonstrate how community counsellors were able to understand and embody the principles of ethical research practice but that, subscribing to these principles emphasised by the research team, limited counsellors’ authority to persuade individuals to conduct an HIVST, while on the other hand the quotes show unreasonable expectations laid on the volunteers and how they were conflicted, thereby raising suspicions of data fabrications.

## Whose failure counts: failing the community?

Table 2 lists the main reasons for defining counsellors as having failed in their role from the perspective of the communities they served. The list clearly demonstrates that community definitions of failure in assessing volunteer counsellor services were drawn from the standpoint of their own perceptions, particularly driven by their awareness of their right to HIV self-testing services. This right to confidentiality and convenience had been promoted as a driving factor behind the development and promotion of the option of HIVST models (Choko et al. 2011). Whilst individuals felt able to self-present to collect the HIVST kit from the counsellors, they were highly aware of their right to confidentiality and any breach of this confidentiality was associated with the counsellors and to them this translated to their definition of failure.

**Table 2.** Community definitions of counsellor failure.

1. Consistently unavailable at home, inability to dedicate more time to the counselling process and inability to provide kits when required.
2. Breaches of confidentiality.
3. Inability to respond in a timely way to concerns and failure to report concerns to the research team.
4. Failure to represent the community to the research team and a lack of respect towards the community they served.

Community members also described the inability to access test kits on demand as a failure on the part of counsellors. This was often due to the unavailability of counsellors in their homes or the counsellor's failure to visit a particular household to offer HIVST services. Decisions to test for HIV are often agonised over for protracted periods of time but once an individual decides to test then they expect to be able to access services immediately: this was, after all, the promise and potential heralded for HIVST as more convenient than other models. If counsellors failed to provide a service when required, given these circumstances and expectations, their failure was often perceived to have inhibited choice.

Familiarity with the HIVST kit and the attraction of quick results, available directly to the individual testing, increased the popularity of HIVST as a model to keep aware of individual status (Kumwenda et al. 2014). This possibility of regular and repeat testing is an additional benefit discussed for HIVST but in the context of the semi-supervised provision of HIVST in an intervention trial, demand outweighed supply and communities complained that they were unable to access more than one HIVST kit in a 12 month period. Rather than situating this decision as one made by those who brought the trial to their community, they blamed the community counsellors for not providing a sufficient number of test kits and for failing to assist them in accessing another, extra test.

## Whose failure counts: failing the institution?

Table 3 lists the main reasons for failure from the perspective of the research team, defined during the 12 month evaluation. Whilst these conditions for failure were ostensibly more objective than those defined by the community, both have in common a refusal to situate expectations laid on the volunteers within the context of the everyday lives of the volunteering community counsellor.

**Table 3.** Institutional definitions of counsellor failure.

1. Failure to meet uptake targets consistently.
2. Failure to follow correct protocol and procedures including erroneous recording of data in registers and on forms.
3. Unavailability of testing when required.
4. Unavailability and failure to attend monthly counsellor meetings with the research team.

The perspective of the research team on failure was clearly defined by the needs of the intervention trial, particularly in reaching quantitative uptake targets for each cluster on HIVST. It is clear that priority from the institutional perspective was defined by the epidemiological objectives of the study and supported through a series of monitoring targets throughout the year before the 12 month evaluation took place. These monitoring mechanisms were in place to monitor progress from the perspective of the project goals but also to assist the counsellors throughout the period to ensure good and ethical research practice and to keep on track to meet the institutional, research-driven goals. Community counsellors were deemed to have failed the research project if at any point of delivering services to community they failed to adhere to externally defined guidelines and procedures. Consistent failure to test the target number of clients was regarded as evidence of a lack of commitment from community counsellors. Other serious breaches of protocol included providing HIVST kits to those under the age of 16 or to those resident outside the intervention cluster. This decision was wholly driven by research needs, in order that the trial avoided contamination across control clusters, necessitating stringent control mechanisms to monitor access. Apart from the two counsellors who had ‘failed’, there was no other evidence of data fabrication and the trial was designed in such a way that fabrication would have been difficult.

In contrast to accessibility failure defined by the community, counsellors were considered to have failed from the perspective of the institution if they were unavailable during pre-defined periods within a day, specifically between 8.30am and 4.30pm with some flexible weekend work. In defining these hours of work there had been a ‘failure’ on the part of the research team to understand underlying community structures and livelihood dynamics of local urban communities.

## Discussion

This ‘evaluation of an evaluation’ has highlighted the constraints under which Community Counsellors work with the need to fulfil conflicting roles in order to satisfy the expectations of multiple audiences; the community and the research institution. They are at one and the same time working for the intervention trial, albeit under a voluntary status, and representing the community with whom they live in providing a valuable and valued service of HIVST. They are at once insiders and outsiders, those who facilitate access and those who restrict access, those who embody the aims of the trial in their practice and those who embody the needs of the community. This interstitial social position between the trial and the community highlights a disconnect between social structures and agency on the part of the individual (Giddens 1984; Bourdieu 1990), and the challenges this raises provides much of the explanation for the ‘failures’ described in the evaluation.

First, community counsellors are charged with and are trained to ensure ethical practice according to the needs of the intervention trial, whilst they are also responsible, and ascribed the responsibility for, ethical implementation of HIV testing and counselling through the offer of HIVST, promoted as a valuable addition to testing modalities largely because it increases access to testing. As ethical practitioners, community counsellors are morally in a position to support and promote access to HIV testing as a human right, and indeed their training as national counsellors emphasises this aspect (Kok and Muula 2013). As volunteer counsellors within the context of the intervention trial, however, they are expected to adhere to the trial protocol, which restricts access to HIVST through its intervention-control design, particularly emphasising cluster-level access, age restrictions and multiple tests for individuals. This places counsellors in an ethically challenging position, forced to negotiate choices under conflicting moral codes, and often leads to the development of their own definitions of ethical practice, ‘enacting their own interpretations of justice and beneficence and exerting their own agency’ in the decisions they make to ensure they meet often conflicting social and ethical expectations (Kingori 2013). This was one of the main challenges they faced and an explanation for ‘failure’ defined both by the community and the research institution. Responding to this challenge and at the same time ensuring ethical and equitable access to the HIVST opportunity with high demand from those within neighbouring clusters (Sambakunsi et al. 2014) was sufficient justification for the counsellors to move beyond the boundaries of their own cluster. At the same time, this provided sufficient rationale for the research team to define them as having failed in their role because they had not conformed to the trial protocol.

Secondly, ‘failed’ counsellors were charged with suboptimal numbers of individuals tested within their clusters by the research institution. This was particularly the case with insufficient numbers of men tested across the cluster. Whilst the development of the HIVST model was informed by formative research, ultimately the intervention demanded its own terms, defining very high numbers actually tested per day (between 8 and 16), without regard for underlying gendered social structures, and defining work patterns for men were not adequately taken into account. As a result, counsellors were unable to satisfy institutional requirements because they needed to satisfy their own income earning needs but similarly they were unable to satisfy community requirements because these gender structures and livelihood dynamics had not been taken into account.

And thirdly, counsellors failed because they were not available throughout the required periods of 8.30am to 4.30pm during the week. They were recruited as volunteers and paid minimal salaries under the expectation that they would supplement this with alternative income opportunities. Whilst their role was defined as voluntary, suggesting part-time, in reality, the research team expected them to be available at home during prescribed hours and days, implying full-time availability. Their pursuit of alternative sources of funding opportunities made sense in the context of their interpretation of the role as part-time; in practice, the recruitment pressures meant that the time that the job took was more than part-time.

Recruitment to the position of community counsellor and specific criteria for selection focused on the need to identify individuals who were known to be dependable, respectful, accessible and honest, suggesting that those selected had a prior relationship with their community that outlived the duration of the trial and that would remain once the intervention had ended. It was not technical competence as such that made successful volunteers, but socio-demographic backgrounds, character and, most importantly, ethical conduct. Assessments of the moral character of counsellors as criteria for selection were made without either researcher or community acknowledgement of the impact this position would likely have on their ability to perform their work whilst maintaining their reputation as moral citizens. The need to maintain social relationships by navigating reciprocity and mutual aid is a priority coping strategy in resource-poor contexts (Desmond 2009). Awareness of these important relationships that would outlive the trial may have influenced those who ‘failed’ through offering tests to social relations outside the cluster, suggesting the emphasis on a moral responsibility beyond the ethical practice promoted through the trial. The selection to the counsellor role by their own communities reinforced this moral responsibility towards those same communities and promoted the moral authority of those who had selected them, over how they managed their role as counsellors.

However, counsellors were also subject to the ethical requirements and demands of their position within the trial, having accepted the terms and conditions of the position when taking it on, despite the fact that these same conditions were in themselves ethically contested, particularly given the ‘full-time voluntary’ nature of their position and the tensions this created in their performance and praxis. Emphasis on adherence to the guidelines prescribed within the intervention trial context, based on international codes of practice and standardised procedures for data collection in research, gave them an additional moral responsibility to the research and its success. Their position between these two different social structures informing their role – that of the research team and that of the community – forced community counsellors to exert their own agency, faced with daily choices in navigating these often conflicting, moral systems. Bourdieu suggested that, in order to reconcile structure and agency, individual actors are socialised within a particular ‘field’, formed by a set of relationships or social roles and these social roles are in turn influenced by underlying social structures and forms of capital (Bourdieu 1990). Counsellors were recruited through these underlying social structures but forced to confront them if they conformed to the expectations of the research. Whose interests were community counsellors supposed to serve as they performed their duties? And whose failure actually counts?

## Conclusions

This paper provides insights into the experience and definitions of failure within research. The findings are relevant for community-based studies involving community volunteers across both health and development fields. They also provide a relevant contribution to our understanding of the ethical issues around frontline workers, particularly in resource-poor settings (Lehmann and Sanders 2007; Molyneux et al. 2010; Takasugi and Lee 2012; Kingori 2013). The paper emphasises the multiple dimensions and interpretations of failure and the responsibilities community counsellors take on when they work as volunteers within an externally driven intervention trial. The tension between the priorities of the community and those of the research and its embodiment within each of the areas of failure described above raises key issues in the design and implementation of intervention trials using voluntary community health workers to supply research-driven services at community-level. They highlight the ethical dilemmas experienced regularly by individuals employed within such roles and the strategies that individuals adopt to negotiate the many unacknowledged contradictions they face in taking on this position.

By defining failure based on the evaluation criteria, did the study itself fail to pursue the meaningful participation it promoted through the process of recruiting community volunteers? There is a need to ensure a reasonable balance between meeting external research targets and operating in an ethical manner within the community. In contradiction to the standardised approach promoted to improve rigour within intervention trials, this sometimes entails a process of negotiation and choice, subject to the ethical decisions made by the agents delivering the intervention. Interventions should be flexible in recognition of this contradiction, particularly when recruiting community-based volunteers to deliver the service. This approach would help to ensure that those charged with community-based delivery, particularly as voluntary community employees, are supported by the intervention to negotiate these contradictions between research objectives and community responsibilities, ‘achieving’ intervention success and reducing their likelihood of failure across both structures.
